# Hospital Meetings, &c.

**Published:** 1899-12-23

**Authors:** 


### The Institutional Workshop.

### HOSPITAL MEETINGS, &C.

### METROPOLITAN HOSPITAL SUNDAY FUND.

The Lord Mayor (Mr. Alderman Newton), as president and treasurer, took the chair at the annual general meeting of constituents of the Metropolitan Hospital Sunday Fund at the Mansion House on Wednesday, 13th instant. Among those present were: The Chief Rabbi (Dr. Adler), Archdeacon Sinclair, Canon Fleming, Canon Pycke, Prebendary Kitto, Prebendary Ridgeway, Rev. Dr. Rigg, Rev. C. H. Grundy, Rev. Walford Green, Rev. A. Harland, Rev. G. R. Thornton, Lord Stamford, Sir Dyce Duckworth, Sir Savile Crossley, Alderman and Sheriff Treloar, and Messrs. W. R. Tidd, F. H. Norman, John Mackrell, G. Drummond, A. L. Cohen, Alfred D. Tripp, M.V.O., F.R.C.S., and J. Astley Bloxam, F.R.C.S.

The report presented for the year ended October 31st stated that the amount fer this, the twenty-seventh year of collection, was £53,504, which compares with £40,397 last year and with £37,394, the average annual receipts since the Fund was established. The Council took the opportunity of congratulating Sir Sydney Waterlow—in whose mayoralty the Fund was inaugurated, and who has ever since taken the leading part in its management—on seeing his hope realised that the grand total of the Fund would this year go over one million sterling, the actual figures being £1,009,638. The collections on Hospital Sunday, June 11th, from congregations within the metropolitan area amounted to £38,194. The Rev. Prebendary C. J. Ridgeway, M.A., of Christ Church, Lancaster Gate, headed the list with £1,375; the Rev. Canon Fleming, B.D., of St. Michael's, Chester Square, collected £1,341; the Rev. J. Storrs, M.A., of St. Peter's, Eaton Square, £1,081; and the Rev. Prebendary Eardley Wilmot, M.A., of St. Jude's, South Kensington, £742. Twelve more congregations had collected this year, and there was an increase of £1,661 from congregational collections. Mr. George Herring had sent the munificent donation of £10,000, expressing his high approval of the manner in which the Fund was administered; 1,000 guineas was received from Mr. J. Lane Densham; Sir Savile Crossley again contributed £500, and also sent £500 to the Prince of Wales's Hospital Fund; and "Delta" sent his twenty-first donation of £200.

Sir Savile Crossley moved the adoption of the report. He congratulated the Council on the amount collected this year, and said it showed that the establishment of a third fund, the Prince of Wales's Fund, had not only done no harm but was doing good both to the Sunday and the Saturday Funds by drawing more attention to the hospitals. The work of the Distributing Committee had grown greater and had been done more thoroughly. They all, he felt sure, associated themselves with the Council in congratulating Sir Sydney Waterlow on the realisation of his hope that this year the grand total of a million would be reached. They regretted his absence that day, but they knew he was abroad, recruiting his strength that he might make further efforts for the Fund. With so many calls upon generosity as there were in these days it was of the utmost importance that the claims of the sick and suffering at home should not be forgotten.

Canon Fleming seconded the resolution. While feeling glad to be among those who headed the list, he hoped it would be remembered that the congregations and ministers who had done least had done their best. He endorsed all that had been said about Sir Sydney Waterlow. There was no man who had done more for the Fund, if so much, as he.

The resolution was put and carried unanimously.

The Chief Rabbi moved the continuance of the laws of the constitution. These laws, he said, had been in force for twenty-seven years, and the necessity had not yet arisen to alter or to modify them, which was due, he thought, to the fact that they were so ably administered by the Council, and that they had so able a secretary. He would make one remark bearing upon the last of them—that 5 per cent. should be appropriated for surgical appliances—that while we were full of admiration for the surgeons and nursing sisters who had gone to South Africa to help our brave soldiers, we must not forget that it was in the hospitals and by the use of surgical appliances that they learnt their craft and were fitted for their work. There was some reason to apprehend that the Hospital Fund would suffer owing to the appeal which the Transvaal War was making on people's hearts and pockets. But while giving again and again amply and largely to the War Fund, we must not forget those who had fallen at home in the struggle for life.

Sir Richmond Cotton seconded the motion, which was adopted.

Prebendary Ridgeway proposed that the Council for the year 1899 be re-elected for 1900, with the Earl of Meath, Sir Trevor Lawrence, Mr. Alderman Bell, and Mr. J. R. Cooper to fill vacancies. He mentioned that Lord Meath had not been asked yet to accept the nomination, but he quite hoped that he would see his way to serve.

This was seconded by the Rev. Dr. Rigg, who said that, having been a member of the Council himself from the very beginning, it was a source of singular satisfaction to him to look back upon their history, which was one of continued progress. At first they had great difficulty in getting the constituent members of this vast unity of London to realise, each in his place, his responsibility towards all the other parts, and regard its needs from the point of view of the whole. Now, however, he thought, all London felt an interest in the success of this Fund, in the great needs which the Fund was intended to meet. This change was largely due to the enthusiasm of a few noble men and to the noble example set by some ministers, clergymen of the Church of England in particular, though no doubt some of the poorest had done their best.

The resolution was carried unanimously.

The Archdeacon of London then proposed that June 24th be fixed for Hospital Sunday of 1900, and that the cordial co-operation of all ministers of religion within the metropolitan area be again invited in the usual way. On their usual date, June 17th, the Society for the Propagation of the Gospel—a society more than 120 years older than theirs, and collecting a much larger amount of money for good work—was celebrating its bicentenary, and the Council and the Bishop of London felt that it was not possible to run counter to it. To other possible dates there were objections, and after all it was only a week later than usual. He was glad to see that £1,661 more had been received from churches and chapels, twelve more congregations having contributed. He hoped some day that all the churches and chapels in the metropolis would take part in the collection without a single exception. As yet not quite all did so, and he wondered whether the circulation privately of the exceptions would have a good effect. He was going to suggest that the Lord Mayor should invite the non-contributing ministers to dinner to talk the matter over, but perhaps that would have the effect of adding to the list instead of lessening it. (Laughter.) He thought that occasion ought not to be let pass without again expressing their thanks both to the *Lancet* and to The Hospital for the help they each year rendered. With the valuable material supplied by those journals it was only necessary for the clergy to write a text and prepare a peroration and exordium, and their sermon for Hospital Sunday was complete. This year they must be more on their mettle than ever before; the drain on their resources would be very great, and they must work not in rivalry, but in co-operation with the War Funds.

Canon Leopold Pycke, in seconding the resolution, remarked that though in matters of religion they might differ as far as pole from pole, yet in working for the poor and for the hospitals they were all absolutely at one.

Mr. Mackrell proposed a vote of thanks to the Lord Mayor for presiding, and the Rev. C. H. Grundy, in seconding this, said someone had spoken of the Mansion House as the Clapham Junction of charity, since from the Mansion House funds went to the ends of the earth in that sacred cause. Lord Mayors might come and go in a sort of apostolic succession, but the Hospital Sunday Fund went on without a break, and each succeeding Lord Mayor seemed better than his predecessor. Referring to Archdeacon Sinclair's suggestion, he thought perhaps if they were to print in their list the names of all the churches and chapels in the metropolitan area, leaving blanks in the money column against those which did not contribute, it might bring about a wholesome feeling and so produce the desired result. And he regretted also to see in their list as it stood that some congregations sent only a part of the offertory received; Hospital Sunday should be devoted throughout London entirely to the Hospital Sunday Fund.

The resolution was adopted, and the Lord Mayor, in acknowledging it, said the suggestions made should at the proper time receive attention. He hoped that by the wave of generosity which was now animating the nation such a broad catholic spirit would be engendered that that Fund might even benefit rather than suffer.

